# Rise of the mutants: report from the 19th conference of the European Haematology Association, Milan, 12–15 June 2014

**DOI:** 10.3332/ecancer.2014.453

**Published:** 2014-08-18

**Authors:** Luca Mazzarella

**Affiliations:** European Institute of Oncology, Via Ripamonti 435, Milan 20141, Italy

**Keywords:** epigenetic, sequencing, targeted therapy, chronic lymphoid leukaemia, acute myeloid leukaemia

## Abstract

At the 19th conference of the European Haematology Association in Milan, we saw the true and dramatic changes brought about by the integration of extensive genomic information in clinical practice, and the dilemmas that accompany such a rapid increase in knowledge. Each disease is sliced more and more into smaller pieces, each with its own better-determined outcome and treatment. We also observed the rise of mutant-specific epigenetic agents, which benefit from knowing the underlying genetic abnormality to specifically assign an epigenetic drug where it is needed. In contrast to the ‘one mutation, one drug’ approach, others are pursuing the search for drugs targeting pathways fundamental for the survival of all or most cancer cells, sometimes looking at more ‘exotic’ pathways like neddylation or nuclear export.

## Introduction

At the 19th conference of the European Haematology Association in Milan, we saw the true and dramatic changes brought about by the integration of extensive genomic information in clinical practice, and the dilemmas that accompany such a rapid increase in knowledge. Each disease is sliced into increasingly smaller pieces, each with its own better-determined outcome and treatment. We also observed the rise of mutant-specific epigenetic agents, which benefit from knowing the underlying genetic abnormality to specifically assign an epigenetic drug where it is needed. In contrast to ‘one mutation, one drug’ approach, others are pursuing the search for drugs targeting pathways fundamental for the survival of all or most cancer cells, sometimes looking at more ‘exotic’ pathways like neddylation or nuclear export.

## Clinical implementation of genomics

Several talks across all disease types reviewed how genomic information is changing our approach to the treatment of patients. In some cases, this bulk of new information can cause real dilemmas when our clearer view of a prognosis associated with a certain patient collides with clinical considerations. These dilemmas are perhaps most strongly felt in the field of chronic myeloid diseases, like CMML, MPNs, and MDS (presented by Mario Cazzola and Ben Ebert), in which the possible outcomes are extremely variegated, ranging from relatively indolent to aggressive diseases, and patients are often elderly and frail. As a result, practitioners are forced to include additional considerations on quality and duration of life. In MDS, for instance, a prognostic score based on routine lab findings (the IPSS-R score) has become established in recent years. However, as Platzbecker provocatively asked, what would one do with a patient with a low/intermediate-risk IPSS-R score, for whom watchful waiting would be the treatment of choice, who bears a mutation in ASXL1 that more than doubles his risk of developing AML [1]? Would we be happy to switch to a more aggressive treatment based on mutational information? And how do we design trials that systematically address these issues? Dilemmas like this accurately describe the difficulty of implementing genetic information in the clinic: the progressive stratification into better-defined prognostic groups makes the design of studies validating the usefulness of that information more difficult using orthodox designs, because possible sample sizes are smaller and harder to define. To cite another example provided by Dr Ebert, the presence of TET2 mutations would suggest a better response to Azacytidine in MDS, but if ASXL1 mutations are present, their negative prognostic effect would be dominant, and azacytidine would not necessarily be the treatment of choice [2]. So genetic information is important, but if incomplete, it might still be useless.

Dr Dohner provided a comprehensive overview of clinical genomics in AML in his Jose Carreras lecture. He illustrated several proposals for revision of the current WHO classification of AMLs, the most important of which was the creation of a new entity defined by RUNX1 mutations. These mutations have specific characteristics, such as more advanced age at diagnosis, worse prognosis, and occurrence in a wide spectrum of myeloid diseases, including secondary AMLs and MDS. He then illustrated several examples of molecular markers driving therapy, mentioning results of a proof-of-principle (AMLSG 11-08) study on the addition of Dasatinib to standard induction and maintenance therapy in CBFb AMLs, in which the drug may be useful because of high frequency of mutations or increased expression of cKIT. Preliminary positive results are now being validated in a randomised trial (AMLSG 21-13).

The discovery of IDH mutations certainly took the AML field by storm [17]. A telling example is the realisation that the oncometabolite D2HG can be used to identify bearers of IDH1/2 mutations and monitor the disease over time [3]. Now, Heilig and colleagues at Heidelberg show that pretreatment levels of D2HG in IDH-mutated patients predict outcome in those enrolled in the AML2003 SAL trial with risk-adapted postremission therapy.

## Personalised epigenetic drugs: preliminary results from the first-in-human trials

One of the most interesting leads at the conference was certainly the appearance of initial results coming from new drugs targeting epigenetic enzymes. Past epigenetic drugs like Azacytidine or Valproate were tested, assuming that a common epigenetic dysregulation was associated with leukaemia. What clearly distinguishes this new breed of drugs and trials is the realisation that epigenetic profiles are dictated by the underlying genetic abnormality. Thus, MLL-mutated AMLs are characterised by alterations in the methylation of a specific histone residue, lysine 79 on histone 3 (H3K79), due to aberrant recruitment of the methyltransferase hDOT1L, but non-MLL mutated are not [4]. IDH-mutated AMLs, conversely, are characterised by specific DNA methylation alterations caused by interference of the oncometabolite D2HG with DNA methyltransferases, and thus bear a specific DNA methylation profile [5] that can be counteracted by inhibiting the mutated enzyme. Finally, the interpretation of the epigenetic mark by reader proteins like those bearing the ‘Bromodomain’ can be targeted and results in the loss of aberrant transcriptional programme and cell death [6]. This sensitivity for BRD4 inhibition seems to stem from a sort of epigenetic addiction to superenhancers characteristic of leukaemia and appears to be a more general feature of cancers, independent from the underlying genetic anomaly [7].

Each of these epigenetic processes can be targeted in an extremely specific way by drugs that have just initiated their first-in-human dose-escalation trials: the anti-hDOT1L drug EPZ-5676 for MLL-mutated AMLs presented by Scott Armstrong (presented at EHA for the first time since study initiation), AG-221 for IDH-mutated AMLs presented by Dr De Botton and the anti-BRD2-4 drug OTX015 presented by Jay Bradner (the latter two both presented earlier this year at AACR). In their clinical translation, these drugs had striking similarities: very low toxicity, such that a maximum tolerated dose could not be identified to date, and a high rate of responses despite previous refractoriness to standard therapies, characterised by an initial wave of blast differentiation reminiscent of ATRA therapy in APL. Targeted epigenetic drugs appear as one of the most promising therapeutic advancements at the moment.

## Other novel compounds

Dr Cortes from MD Anderson reported on the development of the beta-catenin inhibitor PRI-724, in phase 1 in AML patients after a first-in-human trial on solid tumours that showed some effect on cancer stem cells. At the completion of the planned dose-escalation phase, the drug showed great tolerability with no identified DLT; signs of efficacy with a reduction in stem-cell associated phenotypic markers like CD44 were observed. The results of the trial are particularly interesting in light of the intriguing work presented by S Kousteni from Columbia University. She discovered that mice expressing an activating beta-catenin in the osteoblasts, an established model of osteopetrosis, showed a skewed myeloid differentiation and features of AML, due to excessive release of the NOTCH ligand Jagged 1 in the HSC niche [8]. Thus, at least in experimental models, AML can be initiated by mutations that do not arise in bone marrow cells at all, and beta-catenin targeting agents may act at least in part by modulating the niche and not the AML cell directly.

Dr Dohner from Ulm reported on the Polo-like Kinase 1 (PLK1) inhibitor Volasertib, now in clinical development in several phase 1–2 trials. PLK1 is selectively expressed in proliferating cells and demonstrated good therapeutic tolerability, with mostly gastrointestinal toxicity, and promising efficacy in phase 2 on heavily pretreated AML patients. The phase 3 trial is ongoing.

Dr Swords from Miami reported on a new class of drugs that targets the recently discovered process of ‘neddylation’, a post-translational modification that affects a limited number of proteins (about 400) important for cell cycle progression and survival of cancer cells and that involves the addition of the Ubiquitin-like peptide NEDD8 [9]. Trials on the NEDD8-activating enzyme inhibitor MLN4924 (Pevonedistat) were completed in both solid and haematological malignances and supported an application to FDA for use as single agent. An ongoing trial is in combination with Azacytidine in elderly, chemo-unfit patients, based on synergism observed in preclinical studies. A promising overall response rate of 60% was observed, with manageable toxicity.

Further along the line of drugs inhibiting ‘unorthodox’ pathways, initial results on Selinexor (KPT-330) were shown in a variety of malignances, both myeloid (AML) and lymphoid (myeloma and DLBCL). Selinexor inhibits exportin1, a nuclear/cytoplasmic transporter that alters the intracellular distribution and overall levels of several tumor-associated proteins, including p53, FLT3, and KIT. The current phase 1 trials were preceded by good preclinical data [10]. Overall, Selinexor showed very good tolerability without identification of an MTD and promising response rates.

Finally, several talks discussed the development of bispecific antibodies, which work as a bridge between a T-cell receptor component (in the cases presented here, CD3) and an antigen enriched on the tumor surface, which confers specificity. Thus, effector T-cells come in close contact with tumor cells and are activated by the antibody binding, resulting in very specific cytotoxicity. Krupka and colleagues showed interesting preclinical efficacy of the anti-CD33/CD3 antibody AMG330 against AML, with particular action on leukaemia stem cells. Dr Nicola Gökbuget and his colleagues presented results from a trial in relapsed/ refractory ALL with the anti-CD19/CD3 Blinatumomab that showed a promising response rate in the range of 40–50%, which is very good considering the unfavourable setting.

## Myeloid neoplasm biology

Intriguing results were presented by David Kent from Cambridge, who studied myeloproliferative neoplasms bearing JAK2 and TET2 mutations and managed to reconstruct which of the two appeared first. Surprisingly, the transcriptional phenotype was different between ‘JAK2-first’ and ‘TET2-first’, leading to the unintuitive conclusion that genetically identical cells behave differently depending on which mutation came first.

Ruud Delwel presented his groundbreaking work that identified the peculiar mechanism behind AML with inv(3)/t(3;3) (a WHO class of its own). Using a clever combination of functional genomics and genome engineering, they demonstrated that the rearrangement repositions an enhancer of GATA2 upstream of the stem cell regulator EVI1 [11]. In one single hit, the rearrangement causes overexpression of EVI1 and haploinsufficiency of GATA2, both leukemogenic alterations. Interestingly, the aberrant transcription could be reversed by pharmacologic Bromodomain inhibition (see above).

Finally, Ben Ebert’s group seems to have uncovered the reason behind the exceptional responses to lenalidomide obtained in MDS patients with 5q deletions, nailing it down to the gene Casein Kinase 1, involved in the deletion.

## Myeloid neoplasms–conventional therapy

Dr Venditti from Rome and Dr Russell from Nottingham presented an interesting debate on whether the good old ‘3+7’ combination of cytarabine and an anthracycline for induction therapy in AML should be improved on by adding or substituting drugs or by modifying the schedule. A long and unresolved debate concerns the use of Ara-C at higher doses, which provides a moderate event-free survival, but little in terms of overall survival, at the price of higher toxicity. Other proposed approaches include the addition of Gemtuzumab Ozogamycin or Retinoic Acid and the Flag-IDA scheme; each has specific advantages and disadvantages and in particular for GO and ATRA, the impact of adding the third drug seems to be dictated by the underlying genetic abnormality.

Along this line, Richard Schlenk presented long-term data from the AMLSG 07-04 trial that evaluated the impact of adding ATRA to risk-adapted induction and consolidation therapy. The authors found a significant advantage for event-free survival and overall survival in favourable risk subgroups of patients, namely those bearing NPM1 mutations without FLT3 mutations and CBFb AMLs.

## Lymphoid biology

Dr Elias Campo and Dr John Gribben presented an interesting overview of our knowledge on the genetics of CLL. Mutations in IgHV play a strong prognostic role, but more recent acquisitions show frequent mutations in genes belonging to a handful of pathways with prognostic relevance (NOTCH, splicing factors, NFKB, and DNA damage response genes). These data can be further refined by adding transcriptomics-based clustering and DNA methylation profiles. Furthermore, targeted therapy can now be envisaged against some of the involved pathways, like NOTCH and splicing factors. An important issue, however, is clonal evolution [12] as the genetic makeup of the disease can vary considerably over time, with the emergence of other genetic drivers, some of which are particularly associated with later-emerging subclones (like KRAS or NRAS, and TP53, which can first appear either at diagnosis or at later stages).

Davide Rossi from Gaidano’s group in Turin identified the transcription factor KLF2 as frequently mutated in splenic marginal zone lymphoma, adding to the genetic subdivision of lymphomas. Incidentally, the result was obtained after a good old-fashioned cherry-picking of candidate genes to screen based on their known function in post-germinal centre B cell biology, showing that the human brain is sometimes still more powerful than brute computational force.

## Lymphoid neoplasms–clinical

Dr Hillmen presented the important results of the RESONATE phase III study comparing daily ibrutinib monotherapy versus the anti-CD20 antibody ofatumumab in relapsed or refractory CLL [13]. At a median follow-up of 9.4 months, ibrutinib significantly improved progression-free survival (PFS); the median was not reached with ibrutinib and was 8.1 months with ofatumumab (hazard ratio [HR], 0.22; *P* < 0.001). Ibrutinib also significantly improved overall survival (HR, 0.43; *P* = 0.005). The effect resisted the planned crossover from ofatumumab to ibrutinib at progression and was regardless of del (17p) or purine analog-refractory disease. Toxicities were manageable. This study consolidates the role that ibrutinib will play in the treatment of lymphpoid malignancies.

John Seymour from Australia presented interesting results on the apoptosis-promoting anti-BCL2 agent ABT-199 in monotherapy in patients with R/R CLL, including del (17p) and fludarabine refractory disease. The overall response rate was high at 77%, and elevated rates were observed also in high-risk patients.

Dr Salles gave a provocative presentation on the road towards chemo-free therapy for follicular lymphoma. There is still limited knowledge about what is the most important cytotoxic mechanism for Rituximab and other anti-CD20 antibodies. ADCC plays a major role, also demonstrated by the variability in response dictated by FCg receptor polymorphisms. Novel anti-CD20 antibodies like Ofatumumab, Ocrelizumab, and Obinutuzumab, which bear structural modifications that attempt to increase direct or cell-mediated cytotoxocity, are under development and could be used together with Rituximab. Other attempts at adding immune-stimulating cytokines had poor results, whereas combination with drugs that directly stimulate T-cell activity had promising results. These include lenalidomide and the anti-PD1 antibody [14]. Interesting preliminary results were also obtained with Tyrosine Kinase inhibitors, especially Idelalisib in monotherapy [15].

Dr Tiacci from Perugia demonstrated the dramatic efficacy of Vemurafenib in hairy cell leukaemia, a disease in which he and his colleagues demonstrated the frequent presence of the BRAF-V600E mutation typical of melanoma [16]. The history of advancements in this disease is very instructive, as a therapeutic breakthrough was brought about solely by an increased understanding of the biology of the disease, and through investigator-initiated trials with no support from industry.

## Abbreviations

CMML: chronic myelomoncytic leukaemia
MPN: myeloproliferative neoplasms
MDS: myelodysplastic syndrome
IPSS-R: international prognostic score system-revised
AML: acute myeloid leukaemia
ASXL1: Additional sex comb-like
TET2: Tet methylcytosine dioxygenase 2
CBFb: Core binding factor beta
cKIT: proto-oncogene c-Kit
RUNX1: Runt-related transcription factor 1
IDH: Isocitrate Dehydrogenase
D2HG: D-2-hydroxyglutarate
MLL: Mixed lineage leukaemia
hDOT1L: human disruptor of telomeric silening-like 1
BRD4: bromodomain-containing protein 4
ATRA: All-Trabs retinoic Acid
PLK1: Polo-like kinase 1
NEDD8: Neural precursor cell expressed, developmentally down-regulated 8
FDA: Food and Drug Administration
DLBCL: diffuse large B-cell lymphoma
FLT3: FLK1-like Tyrosine kinase 3
MTD: maximum tolerated dose
ALL: acute lymphoid leukaemia
JAK2: Janus Kinase 2
GATA2: GATA transcription factor 2
EVI1: ecotropic viral integration site 1
WHO: World Health Organization
Ara-C: Cytarabine
Flag-IDA: idarubicin, fludarabine, cytarabine, G-CSF)
NPM1: Nucleophosmin 1
GO: Gemtuzumab Ozogamycin
IgHV: immunoglobulin heavy chain variable
NFKB: Nuclear Factor Kappa b
KRAS: K-RAS oncogene
NRAS: N-RAS oncogene
KLF2: Kruppel-like factor 2
FCg: Fragment crystallisable gamma
BRAF: B-Raf oncogene
